# Genetic Variation of the *IL-28B* Promoter Affecting Gene Expression

**DOI:** 10.1371/journal.pone.0026620

**Published:** 2011-10-25

**Authors:** Masaya Sugiyama, Yasuhito Tanaka, Takaji Wakita, Makoto Nakanishi, Masashi Mizokami

**Affiliations:** 1 The Research Center for Hepatitis and Immunology, National Center for Global Health and Medicine, Ichikawa, Chiba, Japan; 2 Department of Biochemistry and Cell Biology, Nagoya City University Graduate School of Medical Sciences, Mizuho, Nagoya, Japan; 3 Department of Virology and Liver Unit, Nagoya City University Graduate School of Medical Sciences, Mizuho, Nagoya, Japan; 4 Department of Virology II, National Institute of Infectious Diseases, Shinjuku, Tokyo, Japan; 5 JSPS Research Fellow, Japan Society for the Promotion of Science, Chiyoda, Tokyo, Japan; Saint Louis University, United States of America

## Abstract

The current standard of care for the treatment of chronic hepatitis C is pegylated interferon-α (PEG-IFNα) and ribavirin (RBV). The treatment achieves a sustained viral clearance in only approximately 50% of patients. Recent whole genome association studies revealed that single nucleotide polymorphisms (SNPs) around *IL-28B* have been associated with response to the standard therapy and could predict treatment responses at approximately 80%. However, it is not clear which SNP is most informative because the genomic region containing significant SNPs shows strong linkage disequilibrium. We focused on SNPs in close proximity to the *IL-28B* gene to evaluate the function of each and identify the SNP affecting the *IL-28B* expression level most. The structures of *IL-28A/B* from 5′ to 3′-UTR were determined by complete cDNA cloning. Both *IL-28A* and *28B* genes consisted of 6 exons, differing from the CCDS data of NCBI. Two intron SNPs and a nonsynonymous SNP did not affect *IL-28B* gene function and expression levels but a SNP located in the proximal promoter region influenced gene expression. A (TA) dinucleotide repeat, rs72258881, located in the promoter region was discovered by our functional studies of the proximal SNPs upstream of *IL-28B*; the transcriptional activity of the promoter increased gradually in a (TA)_n_ length-dependent manner following IFN-α and lipopolysaccharide stimulation. Healthy Japanese donors exhibited a broad range of (TA) dinucleotide repeat numbers from 10 to 18 and the most prevalent genotype was 12/12 (75%), differing from the database (13/13). However, genetic variation of *IL-28A* corresponding to that of *IL-28B* was not detected in these Japanese donors. These findings suggest that the dinucleotide repeat could be associated with the transcriptional activity of *IL-28B* as well as being a marker to improve the prediction of the response to interferon-based hepatitis C virus treatment.

## Introduction

A novel group of cytokines was discovered simultaneously by two independent groups in 2003 and named interferon lambda (IFN-λ) [1], [2] or type III IFN. Type III IFN comprises three members, IFN-λ1, 2, and 3 or *IL-29* and *IL-28A*, and *IL-28B*, respectively. Type III IFN is a member of the class II cytokine family. This family includes type I, II, and III interferons and the IL-10 family (IL-10, IL-19, IL20, IL-22, IL-24, and IL-26). IFN-λ uses a distinct receptor complex consisting of a unique subunit, named IFN-λR1, and the IL-10R2 subunit. Expression of the IFN-λR1 receptor subunit is highly restricted, whereas the type I IFN receptor complex and the IL-10R2 receptor were detected in most cell types [1], [2], [3], [4], [5], [6]. The IL-10R2 receptor subunit is shared by IL-10, IL-22, IL-24, IL-26, and IFN-λ. This suggests that type III IFNs act in a rather cell-type specific manner to mediate their biological functions. Type III IFNs trigger a type I IFN-like gene expression profile [5], [6], [7], which has been shown to have antiviral activity *in vitro* and *in vivo* [1], [2], [5], [6], [8]. Thus, the two types of IFN seem to have similar biological effects at a cellular level. IFN-α and IL-29/28A treatment reduced the concentration of hepatitis C virus (HCV) plus-strand RNA in an *in vitro* assay [6], [9], [10], [11]. In addition, IL-29 may have therapeutic value against chronic viral hepatitis in human patients [5].

Recently, a genome-wide association study (GWAS) revealed that several highly correlated common single nucleotide polymorphisms (SNPs), in a linkage disequilibrium (LD) block encompassing the *IL-28B* genes on chromosome 19q13, are implicated in the response of chronic hepatitis C (CHC) patients to pegylated IFN-alpha (PEG-IFNα) and ribavirin (RBV) [12], [13], [14]. The CC genotype of rs12979860 and TT genotype of rs8099917 are associated in CHC patients with a sustained viral response (SVR) of 2.5 or greater rate, which is dependent of ethnicity, compared to the other genotypes. Moreover, the CC genotype of rs12979860 and TT genotype of rs8099917 favor spontaneous clearance of HCV [15].

We have reported the genomic analysis of approximately 15 kb containing the significant SNPs using Haploview software for LD and haplotype structure [14], [16]. To analyze the difference in LD pattern between races, we performed LD mapping with these SNP**s** on JPT (Japanese in Tokyo), CEU (Utah residents with ancestry from Northern and Western Europe) or YRI (Yoruba in Ibada, Nigeria) populations. These SNPs were in strong LD in JPT and CEU populations, although relatively low LD was predicted in the YRI population [14], [16], suggesting that any of the SNP**s** located in this region could be responsible for treatment response. Because of the strong LD, tests for independence among these variants were not able to reveal which of these SNPs is uniquely responsible for the association with virological response (VR) or non-virological response (NVR). The identification of the primary genetic variant located in the LD block remained critical, although the risk haplotype tended to influence the expression levels or activity of *IL-28B* [13], [14]. In this study, we sought to determine the primary SNP affecting IL-28B expression and/or its function by focusing on the proximal regulatory region of *IL-28B*.

**Figure 1:**
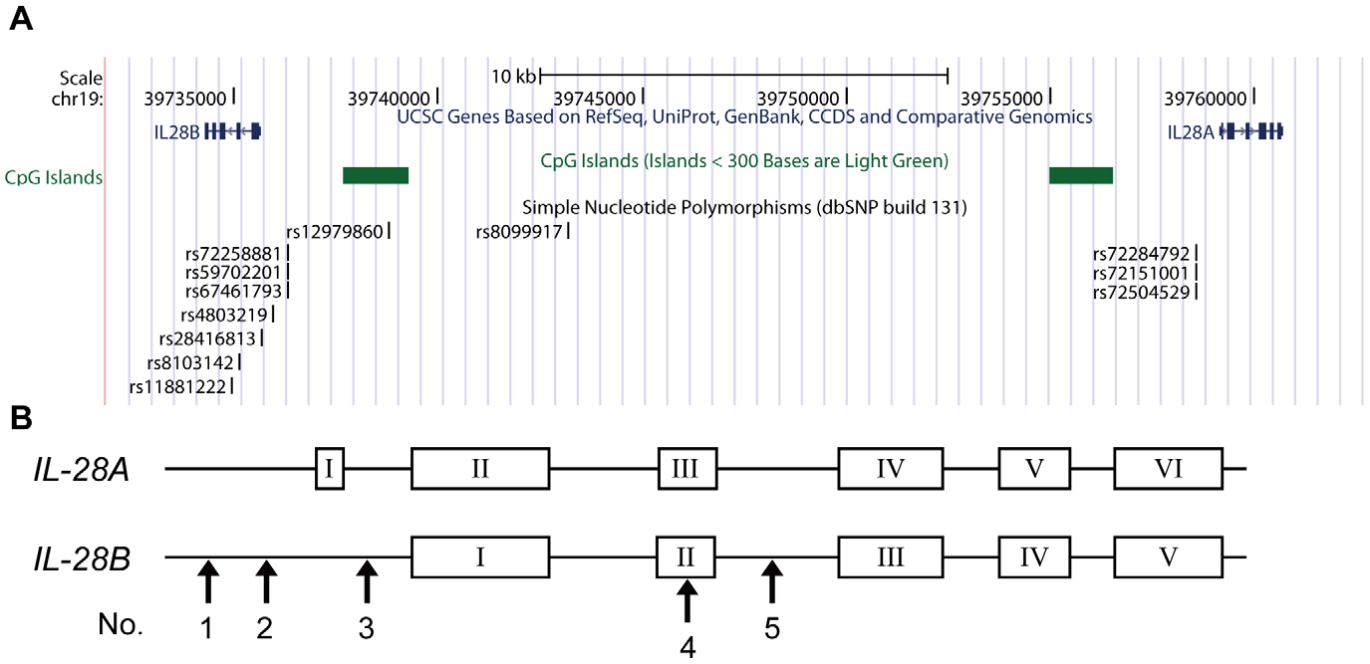
The position of significant SNPs and *IL-28A/B* in chromosome 19, retrieved from the database. (A) The *IL-28A/B* genes located in chromosome 19q13 are described in the genome map of the UCSC genome browser. The significant proximal SNPs around *IL-28B* associated with response to PEG-IFN/RBV therapy are shown in the map [14]. SNPs of (TA)_n_ variation at the regulatory region of *IL-28A* are displayed in the position corresponding to that of *IL-28B*, which is not associated with anti-HCV therapy. (B) The schematic of *IL-28A/B* gene structure is described based NCBI CCDS data. Arrows show five significant SNPs examined in this study (see Table 1).

*IL-28B* was discovered as a member of the IFN-λ family by Sheppard et al. and Kotenko et al. [1], [2]. They discovered this family, *IL-29*, *IL-28A*, and *IL-28B* and the specific receptor, *IL-28R1*, by applying individual computational techniques to the draft human genome. However, the start codon of IFN-λ differs between the reports, with an additional 12 nucleotides at the N-terminus in all IFN-λs reported by Sheppard et al. (Fig. S1). The sequence similarity between these ORFs is approximately 96.7% and, especially, there is a high degree of identity between *IL-28A* and *IL-28B* cDNA (approximately 98%). Figure 1A shows the locations of *IL-28A/B* gene, the significant SNPs around *IL-28B* related to anti-HCV therapy reported in previous studies [12], [13], [14], and (TA)_n_ repeats in the regulatory region of *IL-28A* and *B*. The SNPs information assessed in this study is summarized in Table 1 and the locations of the SNPs are shown in the schematic of the *IL-28B* gene (Fig. 1B). The reference sequences of *IL-28A* or *IL-28B* cDNA, registered in NCBI CCDS, are composed of 6 exons and 5 exons, respectively (Fig. 1B). Because high sequence similarity was observed between *IL-28A* and *IL-28B* from CpG to the region downstream of 3′-UTR (Fig. S2), the genes were almost completely identical around transcription start site (TSS) (>99%). Then, we determined the likely gene structure using a complete cDNA cloning method because a similar transcriptional mechanism was expected for *IL-28A* and *IL-28B*.

**Table 1:** Significant SNPs around *IL-28B*.

Feature	rs ID	Allele 1/2*1	Minus strand*2	Location	No.
DIP*3	rs72258881*4	ATAT/-	TATA/-	Regulatory	1
Substitution	rs4803219	C/T	G/A	Regulatory	2
	rs28416813	C/G	G/C	Intron	3
	rs8103142	T/C	A/G	Nonsynonymous	4
	rs11881222	A/G	T/C	Intron	5

*^1^These data were derived from dbSNP. Allele 2 is the risk allele of HCV therapy reported by Tanaka *et al*., except for rs72258881.

*^2^Complementary nucleotides are shown because *IL-28B* is coded on the minus strand.

*^3^DIP: deletion/insertion polymorphism.

*^4^The ID represents rs72258881, rs59702201, and rs67461793 because these three are located in the same genomic region, the TA repeat.

## Materials and Methods

### Genome samples

Genome samples were obtained from 20 healthy volunteers (HV). Peripheral blood mononuclear cells (PBMC) collected from HV were isolated using the BD Vacutainer CPT Method (BD Biosciences). Genomic DNAs were extracted by standard methods. SNPs were selected from the database at GWAS database (https://gwas.lifesciencedb.jp/cgi-bin/gwasdb/gwas_top.cgi). Written informed consent was provided by all participants in the genotyping study following procedures approved by the Ethical Committee at Nagoya City University.

### Cell lines

Human hepatocellular carcinoma cell lines, HepG2 and HuH7, human hepatocyte cell lines, HuSE2 (kindly provided by Dr. Hijikata in Kyoto University), and the human cervical cancer cell line, HeLa (obtained from The American Type Culture Collection), were cultured in Dulbecco's modified Eagle's medium supplemented with 10% (v/v) fetal bovine serum, 100 U ml^−1^ penicillin and 100 mg ml^−1^ streptomycin. Human leukemia virus type 1 transformed cell line, MT-2 (a gift from Dr. Ueda in Nagoya City University), Burkitt lymphoma cell line, Raji, and human T cell leukemia cell line, Jurkat (obtained from The American Type Culture Collection), were cultured in RPMI 1640 medium supplemented with 10% (v/v) fetal bovine serum, 100 U ml^−1^ penicillin and 100 mg ml^−1^ streptomycin. All incubations were performed at 37°C in a 5% CO_2_ gassed incubator. Recombinant human IFN-λ2 and -3 were purchased from R&D Systems (Abingdon, UK). Natural human IFN-α was purchased from Hayashibara co. ltd. (Okayama, Japan). The mRNA expression levels of receptors stimulated in this study were confirmed by PCR using gene specific primer (Table S1 and Fig. S3),

### Plasmid Construction

As a T/G heterozygote genome of rs8099917 with a strong LD was used as the PCR template, amplicons from the major and minor alleles were obtained for the assay described below. PCR was carried out to amplify the fragment from −858 nt of the ATG site to TGA of *IL-28B*, and the products were inserted into pcDNA3.1/Hyg (pcDNA/MA or mi) or pcDNA3.1/Hyg vector deleting CMV promoter (pdCMV/MA or mi). A FLAG sequence was conjugated to 6^th^ exon, removing the stop codon, for real time PCR analysis. The promoter region from nucleotide position −858 to +30 of *IL-28B* was amplified using pdCMV/MA or mi vector and inserted into pGL4 vector for the luciferase assay. A vector with an antisense insert was prepared as a control. For expression constructs, the wild type (WT) plasmids, pcDNA3.1/wild expressing human IL-28B, and pcDNA3.1/ns-mut expressing human IL-28B harboring a K^74^R mutation, were generated using pcDNA3.1/V5-His-TOPO® (Invitrogen, San Diego, CA) and were used in the subsequent transfections. In addition, pcDNA3.1/AS expressing antisense strand of IL-28B was constructed as a control. We also obtained a pISRE-luc plasmid (provided by Sakamoto N., Tokyo Medical Dental University, Tokyo, Japan). The pGL4.74 vector encoding Renilla Luciferase was purchased from Promega (Madison, WI). These primer sequences are available on request. The above expression vectors were modified for the analysis of splicing function by introducing two intron SNPs (rs28416813 and rs11881222) (Table 1), which were pcDNA/WT, d-iSNPs.

### Transient transfections

Transient transfections of HeLa, Jurkat, Raji, HuH7, HepG2, or HuSE2 (hepatocellular carcinomas cell line) cells were carried out using FuGene HD (Roche) or the Cell Line Nucleofector kit (Amaxa Biosystems) according to the manufacturers' protocols. Briefly, Cells (2×10^5^) were seeded into a 6 well plate and transfected with for FuGene HD. For the electroporation method, cells (1.0×10^6^) were collected and resuspended in Nucleofector solution V for each individual transfection sample.

### 5′-, 3′-RACE based on full-length cDNA cloning

Total RNA was prepared from cell lines stimulated with lipopolysaccharide (LPS) (0127:B8, Sigma-Aldrich) for 4 hours after 100 U/mL of IFN-α for 16 hours by following previous paper [17]. A GeneRacer Kit (Invitrogen Life Technologies) was used to obtain the complete cDNA sequence of *IL-28A/B* following manufacturer's instructions. Briefly, the GeneRacer RNA Oligo was ligated to the 5′ end specifically of full-length mRNA within the total RNA mixture. This ligated mRNA was then converted to cDNA using reverse transcriptase (RT) and the GeneRacer Oligo dT Primer. Next, this cDNA was used for PCR using the oligonucleotides of GeneRacer 5′ Primer and P1 primer which hybridized to the coding strand of the *IL28A/B* (Table S1). The resulting PCR products were then used for a second round of PCR using the oligonucleotides GeneRacer 5′ Nested Primer, which represents the DNA equivalent of the 3′ end of the GeneRacer RNA Oligo, and P2, which hybridizes to the coding strand of the *IL-28A/B* 5′ to the P1 hybridization site. For 3′ RACE, the cDNA was subjected to the polymerase chain reaction (PCR) to amplify the 3′ end using a forward gene-specific primer P3 designed from *IL-28A/B* and the GeneRacer 3′ primer provided with the kit. Nested PCR, using the same gene-specific primer and GeneRacer 3′ nested primer, was performed. The PCR product of 5′ and 3′ RACE was cloned into pCR4-TOPO TA vector according to the manufacturer's instructions (Invitrogen). Ten clones were isolated and subjected to automated sequencing (ABI3100, ABI) in our core facility.

### Protein expression and purification

Recombinant IL-28B and its mutant were produced by transfecting Free-Style™ 293-F cells (purchased from Invitrogen, Carlsbad, CA) with the expression plasmid, which was grown in 5000 ml of FreeStyle 293 Expression Medium, following the manufacturer's recommendations (Invitrogen, Carlsbad, CA). Cultures were maintained at >90% viability on a shaker plate (Titer Plate Shaker; Lab-Line Instruments, Melrose Park, NJ) moving at 125 rpm in a 37°C incubator with 8% CO_2_ and subculturing at a 1∶10 ratio upon reaching a density of 2×10^6^ cells per ml. Cell density and viability were evaluated with a hemocytometer using 0.4% trypan blue staining. After 96 h, the transfected cell culture was harvested. The **s**upernatant containing the secreted recombinant protein was centrifuged (100×g, 15 min), frozen, and stored at −30°C until use. The 293-F cells supernatant containing the recombinant protein was loaded onto a Ni^2+^ column (Amersham Biosciences) following the manufacturer's directions. Fractions were eluted with 80, 100, 250, and 1000 mM imidazole (in 50 mM Tris, 300 mM NaCl, pH 8.0), and the fraction eluted at 250 mM was pooled and concentrated in an Amicon (10 kDa molecular weight cutoff) to 1 ml (Amersham Biosciences).

### Western blot analyses

Purified recombinant protein was loaded on**to** 12% sodium dodecyl sulfate gels. Proteins were detected with goat anti-IL28 (1∶2000) polyclonal antibody (Santa Cruz Biotechnology, Santa Cruz, CA) and the secondary antibody. Proteins were visualized using ECL Plus Western blotting detection reagents (GE Healthcare) and a LuminoImager (LAS-3000; Fujifilm). The band densities were analyzed with the Multi Gauge software (version 3.1; Fujifilm).

### *IL-28A/B* promoter genotyping

Germ-line DNA was extracted from PBMC according to standard methods [14]. Twenty HV samples were genotyped for the dinucleotide insertion/deletion (indel) present in the promoter region of *IL-28A* or *B*, as described below. Twenty ng of genomic DNA were subjected to PCR analysis in 50 µl aliquots containing 20 pmol of each primer, 5×PrimeSTAR GXL Buffer, 2.5 mM each deoxynucleotide triphosphates, and 1.25 units of PrimeStar GXL DNA polymerase (TAKARA Bio Inc, Tokyo, Japan). The primer pair, G1 and G2 (listed in Table S1), was used for the simultaneous amplification of the *IL-28A* and *28B* regulatory regions. The PCR conditions were as follows: 30 cycles of 10 s at 98°C, and 120 s at 68°C in addition of initial denaturation at 98°C for 5 min and a final extension at 68°C for 10 min. To separate the *IL-28A* amplicon from that of *IL-28B*, 10 µl of PCR products were analyzed using agarose gel electrophoresis and extracted with QIAquick Gel Extraction Kit (Qiagen). Each extracted product was analyzed by direct sequencing using Seq1 and Seq2 primers (Table S1). For further testing of the TA repeat, heterozygous samples were cloned into the pGEM-Teasy vector to count the number of TA repeats in each allele. Six clones were isolated and subjected to sequencing analysis using the primers described above.

### Reporter assay

Luciferase assays of recombinant protein were performed using Dual-Glo Luciferase reporter assay system (Promega, Fitchburg, WI). In toll-like receptor (TLR)-stimulated experiments Raji cells were transfected and left for 16 h with 100 U/mL of IFN-α, then were exposed to LPS (3 µg/ml) for 4 h before harvesting. For assessments of recombinant protein, HeLa cells were transfected with pISRE-Luc and pGL4.74, and were harvested 24 h after IFN-α or λ treatment. The chemiluminescence was measured by SpectraMax L (Molecular Devices, Sunnyvale, CA). Firefly luciferase activity was normalized to Renilla activity to adjust for transfection efficiency.

### Real-time PCR detection

Jurkat cells were transfected with the *IL-28B* expression vector harboring a FLAG sequence derived from the natural promoter (pdCMV/MA, mi, or AS). To induce IL-28B expression, TLR and IFN-α stimulation was given as described above. FLAG and glyceraldehyde-3-phosphate dehydrogenase (GAPDH) mRNA expression were measured using a real-time PCR performed on ABI Prism 7700 sequence detection system (Applied Biosystems) using primer sets (Table S1) after total RNA extraction and reverse transcription (RT) using an RT kit and TaqMan Universal PCR master mix (both Applied Biosystems), according to the manufacturer's manual. Relative gene expression was calculated as a fold induction compared to the control. Data were analyzed by the 2[-Delta Delta C(t)] method using Sequence Detector version 1.7 software (Applied Biosystems) [18] and were normalized using human GAPDH. A standard curve was prepared by serial 10-fold dilutions of human cDNA or FLAG plasmid. The curve was linear over 7 logs with a 0.998 correlation coefficient.

### Statistical Analysis

Statistical analyses were conducted by using SPSS software package (SPSS 18J, SPSS, Chicago, IL) and Microsoft Excel 2007 (Microsoft co., Redmond, WA). Discrete variables were evaluated by Fisher's exact probability test. The P values were calculated by two-tailed student's t-tests for continuous data and chi-square test for categorical data, and those of less than 0.05 were considered as statistically significant.

## Results

### The identification of IL-28B gene structure

**Figure 2:**
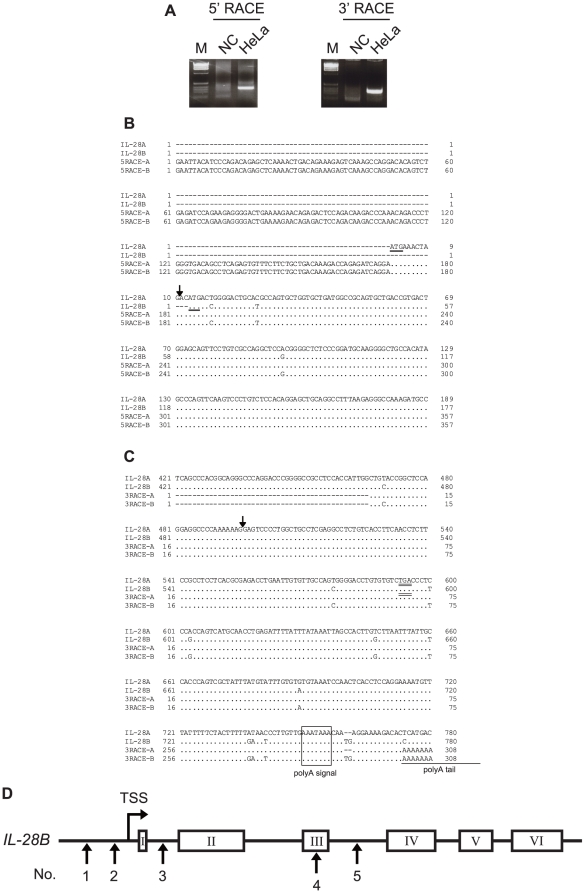
The determination of *IL-28B* gene structure and UTR region. *IL-28A/B* cDNA was isolated using a complete cDNA cloning method and the entire sequences were determined using HeLa, MT-2, and Raji call lines and PBMC from healthy volunteers. (A) 5′- and 3′-RACE analyses were used to determine the complete sequence of *IL-28A/B* mRNA after LPS stimulation (3 µg/mL) for 4 h following IFN-α treatment (100 U/mL) for 16 h. A representative example of agarose gel electrophoresis is shown for the non-stimulated control (NC). PCR products were inserted into the cloning vector and 6 clones of 5′- and 3′-RACE were analyzed by sequencing. (B) mRNA sequences of the 5′ terminal region were aligned using CCDS retrieved from NCBI and RACE data of *IL-28A/B*. The upper two sequences are reference sequences from the NCBI CCDS and the lower two are representative sequence**s** of *IL-28A* and *28B* obtained from 5′-RACE. The underlined triplet indicates the start codon of each gene and arrow shows the splice junctions. (C) mRNA sequences of the 3**′** terminal region were aligned using CCDS retrieved from NCBI and RACE data from *IL-28A/B*. The double-underlined triplet indicates the stop codon of each gene and arrows show the splice junctions. The polyA signal and representative site of polyadenylation also are shown. (D) The derived gene structure of the *IL-28B* is shown with the significant SNPs. The location of SNP No. 3 was changed from the regulatory to an intron region. The transcription start site (TSS) is found behind SNP No. 2.

To define the human *IL-28A* or *IL-28B* gene structure, 5′-RACE and 3′-RACE were performed on total extracted RNA from HeLa, MT-2, Raji, HuH7 cells, and PBMCs from healthy volunteers (Fig. 2A). The sequences obtained matched the genomic contig of AC011445, which contain**s** the sequence of *IL-28A* and *IL-28B* in forward and reverse orientation**s**, respectively. All intron/exon junctions conformed to the canonical GT-AG rule. After stimulation of cells with LPS (3 µg/ml) for 4 h following IFN-α treatment (100 U/mL) for 16 h, *IL-28A/B* transcripts were detected in RACE experiments, but these were not detected in unstimulated cells. The representative TSSs are shown in Fig. 2B and showed little variation among cloned mRNA transcripts. The same 3′-UTR fragment also was detected without any intron in the 3′-RACE experiments (Fig. 2C). A polyadenylation signal (AAAUAAA), located in the 3′-UTR, was found upstream of the polyadenylation site in all samples. All sequences from the transcripts were aligned on the 5′-UTR, the six exons, and the 3′-UTR region of *IL-28A/B*. No different mRNA transcripts of *IL-28A/B* were found in our experiment. Taken together, the *IL-28B* gene structure comprised six exons (see Fig. 2D), and the location of SNP no. 3 (rs28416813) is in an intron, rather than a regulatory region (Table 1).

### The effect of regulatory SNPs on promoter activity

**Figure 3:**
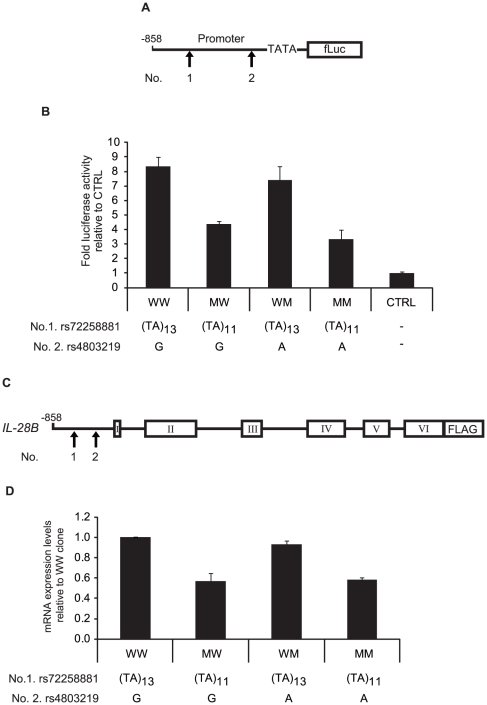
Transcriptional activity of the *IL-28B* promoter region compared between major and minor alleles. (A) The pGL4 reporter plasmid was constructed by subcloning the *IL-28B* promoter subfragment (nt −858 to +30). The combinations of two regulatory SNPs (rs72258881 or rs4803219) were introduced into the pGL4 vector (pGL4/WW, MW, WM, and MM). (B) Raji cells were co-transfected with pGL4 plasmids (0.05 g), and pGL4.74 control plasmid (0.05 g), and tested for firefly as well as renilla luciferase after LPS stimulation (3 µg/mL) for 4 h following IFN-α treatment (100 U/mL) for 16 h. These cells were seeded in a 96-well plate at 10^4^ cells/well. The luciferase activities were normalized with renilla activities and data are presented as fold induction from activation of control vector. Bars indicate the means ± SD of triplicate determinations and the results are from one of three experiments. Statistical analyses are shown in table S2 to avoid complication. (C) For real-time PCR, the combinations of two regulatory SNPs (rs72258881 or rs4803219) were introduced into the pdCMV vector harboring a FLAG sequence (pdCMV/WW, MW, WM, and MM). (D) Jurkat cells were co-transfected with pdCMV plasmids (0.05 µg) and secreted alkaline phosphatase (SEAP) control plasmid (0.05 µg) and the expression levels were quantified using specific primer after LPS and IFN-α stimulation. The FLAG expression levels were normalized with SEAP activities and GAPDH as described in method section. Data are presented as fold induction from expression levels of pdCMV/WW. Bars indicate the means ± SD of triplicate determinations and the results are from one of three experiments. Statistical analyses are shown in table S3 to avoid complication.

Because the TSS was upstream of the position described in previous reports (Fig. 2), two rSNPs (rs72258881 and rs4803219) in the regulatory region were more specifically located in the TSS. A luciferase reporter approach was used to assess the effect**s** of the two rSNPs on promoter activity. Luciferase vector**s** harboring the rSNPs were constructed and used for transfections (Fig. 3A). The promoter activities of the constructs were measured after stimulation with LPS (3 µg/ml) for 4 h following IFN-α treatment (100 U/mL) for 16 h. The transcriptional activity of constructions harboring the (TA)_11_ mutation was reduced (Fig. 3B). Substitution in the rSNP (rs4803219) showed little effect on the transcriptional activity, whereas the number of TA repeats could be responsible for the putative region controlling basal transcription. To confirm the transcriptional activity, Jurkat cells were transfected with full length constructs expressing the FLAG sequence under the control of the natural promoter (Fig. 3C). To avoid the detection of endogenous mRNA, the mRNA with the FLAG sequence was specifically detected by real time PCR using the FLAG primer. The constructs harboring (TA)_11_ yielded lower expression levels after IFN-α and LPS stimulation (Fig. 3D), suggesting that the length of TA repeat in the regulatory region of *IL-28B* could affect the regulation of *IL-28B* transcription.

### Two intron SNPs located near the branch site of splicing

**Figure 4:**
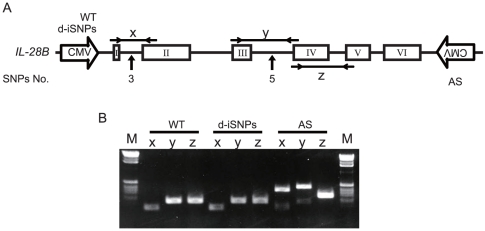
The determination of intron SNPs located near the branch site of splicing. (A) The expression plasmid of WT, d-iSNPs, or antisense (AS) derived from the CMV promoter was transfected into HeLa cells. Schematic of the WT, d-iSNPs, or AS used in the transfection experiments. PCR primers were designed to amplify products between exons. The effect of No. 3 and 5 SNPs (rs28416813 or rs11881222) on splicing were examined by amplicon**s** x and y, respectively. The amplicon z was used for a splicing control. (B) Isolated RNAs were amplified by RT-PCR. The amplified products were checked by 2% agarose gel electrophoresis. The bands from the AS plasmid transcribing antisense represented abnormal splicing of mRNA as a control. Results shown are representative of three independent experiments.

To determine the effect of the two iSNPs on pre-mRNA splicing, HeLa cells were transfected with wild type (WT), a construct with a double mutation of the iSNPs (d-iSNPs), or an antisense (AS) plasmid driven by the CMV promoter (Fig. 4A). The construct providing antisense transcription controlled by the CMV promoter was used to control for splicing defects (AS). Transcripts were analyzed by RT-PCR using primers in exon 1–2, 3–4, and 4–5. The RNA isolated from the WT and d-iSNPs yielded a single band using the three primer pairs. In contrast, longer amplicons were generated in cells expressing the antisense construct (Fig. 4B). The PCR products were sequenced to confirm the origin of the aberrant splicing events derived from the antisense construct (data not shown). The sequence analyses confirmed that PCR product**s** from the WT and d-iSNPs were generated by normal splicing, suggesting that these two intron SNPs resulted in no splicing defects under these conditions.

### No effect of nonsynonymous SNPs on IL-28B function

**Figure 5:**
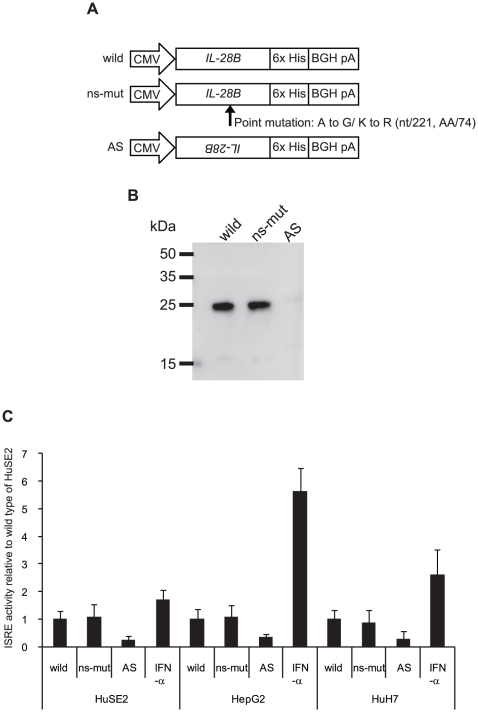
The purification and the activity of recombinant IL-28B with or without nsSNP. (A) The 6×His-tagged expression plasmid of wild type, ns-mut, or AS controlled by the CMV promoter was transfected into 293F cells. Schematics are the wild type, ns-mut and AS used in the transfection experiments. The procedure for recombinant protein purification is described in the materials and methods section. (B) The purified products were confirmed by immunoblotting using anti-IL28B antibody and the secondary antibody. The prepared proteins were loaded onto a 12% polyacrylamide gel. Bands corresponding to the expected molecular weight of IL-28B were observed in the wild type and ns-mut lanes. (C) For luciferase assay, HeLa cells were seeded into a 96-well plate at 10^4^ cells/well and transfected with pISRE-Luc and pGL4.74 control vector before 16 h of IFN-α or IL-28B stimulation. Five ng/mL of IL-28B wild or ns-mut was added to the culture medium. Flow-though liquid from AS expression was used as a negative control. IFN-α (100 U/mL) was added for positive control of ISRE activity. The luciferase activities were normalized with Renilla activities and data are presented as fold induction from the basal promoter activation of the wild type. Bars indicate the means ± SD of triplicate determinations and the results are from one of three experiments.

A nonsynonymous SNPs (rs8103142) located in the 3**^rd^** exon (Table 1 and Fig. 2D) led to the amino acid substitution K^74^R (Fig. 5A). Interestingly, the amino acid at this position is almost always arginine in homologous mammalian IFN-λs (e.g. human IL-28A, mouse IL-28A/B, and rhesus IL-28A/B). Then, the K^74^R substitution was expected to change IL-28B activity. The purified recombinant IL-28B protein (wild type) and the variant (ns-mut) were recognized by anti-IL-28B polyclonal antibody in a western blot assay (Fig. 5B). Based on spectrophotometric measurement of the protein concentration of the eluted fraction, it was calculated that at least 360 µg/mL of purified recombinant IL-28B protein (wild type and ns-mut) was obtained after purification. Flow-through liquid without recombinant protein was provided in the column preparing the sample of pcDNA3.1/AS (Fig. 5B). Molecular processing of IL-28B protein was confirmed to determine the precise N-terminal amino acid by peptide sequencer as the processing site of signal peptide was predicted by computer simulation (http://www.uniprot.org/uniprot/Q8IZI9). Then, the N-terminal sequence, VPVAR, was obtained (data not shown), suggesting that the simulation data was consistent with the form of physiological protein.

To evaluate the effect of nsSNPs on ISRE activity, three hepatoma cell lines (HuH7, HepG2, and HuSE2) expressing IL-28R1 and IL-10R2 were transfected with pISRE-Luc and pGL4.74. These recombinant proteins were added to the supernatant (5 ng/mL each). As shown in Fig. 5C, ISRE activity of the ns-mut protein was similar to that of wild type protein in each cell line. IFN-α (100 U/mL), as a positive control of ISRE activity, showed a strong ISRE activity. These results suggested that the nonsynonymous mutation of rs8103142 did not affect IL-28B activity *in vitro*.

### The genetic variation of TA repeats at the upstream of *IL-28B*

The reference sequence (RefSeq) of the human genome in the international database registers the TA repeat SNPs, rs72284729 or rs72258881, in the regulatory regions of *IL-28A* and *IL-28B*, respectively. The registered basal number of (TA)_n_ is 8 in the regulatory region of *IL-28A* on the plus strand, whereas that of *IL-28B* is 13 on the minus strand encoding the gene (Table 2). From 20 Japanese healthy volunteers, genomic DNA was extracted to determine the actual (TA)_n_ number located in the region of *IL-28A* or *IL-28B* by direct sequencing and, when direct sequencing chromatographs of (TA)_n_ heterozygotes showed mixed patterns from the end of the TA repeat (Fig. S4), the mixed samples were subjected to cloning analysis. Interestingly, the (TA)_n_ number in *IL-28A* was consistently different from dbSNP data, whereas that of *IL-28B* showed varying numbers along with SNPs data. The (TA)_n_ range of *IL-28B* was from 10 to 18, and the most prevalent genotype was 12/12 (75%) in healthy Japanese volunteers.

**Table 2:** The variations of TA repeat in *IL-28A* and *28B*.

		Location	
Gene	Data	rs72284792*1	rs72258881
*IL-28A*	RefSeq. (hg19)	(TA)_8_	
	Cloning	(TA)_8_	
*IL-28B*	RefSeq. (hg19)		(TA)_13_
	Cloning		(TA)_10–18_

*^1^The ID represents rs72258881, rs59702201, and rs67461793 because these three are located in the same genomic region, the TA repeat.

**Figure 6:**
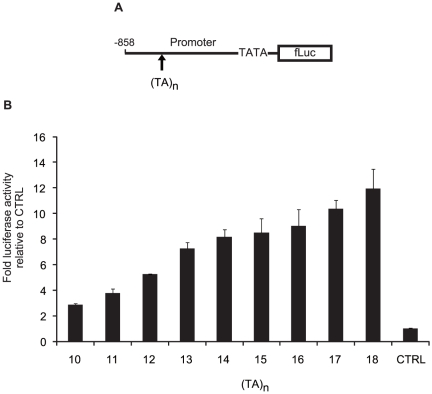
Luciferase assay of (TA)_n_ number. (A) *IL-28B* promoter subfragment (nt −858 to +30) modifying (TA)_n_ number from 10 to 18 was constructed in the pGL4 vector. (B) Raji cells were co-transfected with pGL4 plasmids (0.05 g), and pGL4.74 control plasmid (0.05 g), and tested for firefly as well as renilla luciferase after LPS stimulation (3 µg/mL) for 4 h following IFN-α treatment (100 U/mL) for 16 h. These cells were seeded into a 96-well plate at 10^4^ cells/well. The luc activities were normalized with renilla activities and data are presented as fold induction from the activation of the control vector. Bars, the means ± SD of triplicate determinations and the results are from one of three experiments. Statistical analyses are shown in table S4 to avoid complication.

To determine the functional significance of the TA indel, the regulatory region from −858 bp to +30 bp modifying the (TA)_n_ number was cloned into the pGL4 reporter vector, transfected into HeLa cells, and assessed for firefly luciferase reporter gene expression (Fig. 6A). These cells were treated with 100 U/mL of IFN-α and 3 µg/mL of LPS. The results indicated that the variation in the (TA)_n_ number at this polymorphic locus differentially regulate**s** transcription. The transcriptional activation of the luciferase reporter gene was increased according to the (TA)_n_ number (Fig. 6B).

## Discussion

Four independent GWAS approach**es** have revealed the significant SNPs associated with response to PEG-IFNα/RBV therapy for CHC [12], [13], [14], [19]. These significant SNPs were found around *IL-28B* but not *IL-28A*. The SNPs found in clinical studies to determine the outcome of HCV therapy were rs12979860 and rs8099917, because they showed the statistical significance in each study [12], [13], [14], [19]. However, several SNPs around *IL-28B* were in strong LD (*r* ^2^>0.96) in JPT and CEU populations, although relatively low LD was predicted in the YRI population [16], and so it might be difficult to determine the most informative SNP [16]. These results suggest that any of the SNP**s** contained in this region could be of predictive value.

As reported in previous studies, transcription of *IL-28A/B* was upregulated in the TT genotype of rs8099917, which was associated with SVR [13], [14], [20], suggesting that the expression levels of *IL-28B* could be one of the key factors to clear HCV under PEG-IFNα/RBV therapy and could also affect spontaneous clearance of acute HCV infection [15]. To elucidate this question, we examined the function of the SNPs around the *IL-28B* gene to identify those SNPs affecting *IL-28B* expression. The new findings are as follows: 1) the gene structure of *IL-28B* comprised six exons in the several cell lines tested, although it was registered as having five exons in the CCDS database of NCBI. 2) The substitution of intron SNPs and non-synonymous SNPs in the *IL-28B* gene did not influence the expression levels or function. 3) Increased numbers of TA repeats in the promoter region of the *IL-28B* gene enhanced the transcription activity and expression level of the *IL-28B* gene. Because administration of IL-28B has been shown to have antiviral effects [21], [22], [23], lower expression of IL-28B might lead to a decrease in this effect.

The locations of two SNPs associated with response to HCV therapy, rs8099917 and rs12979860, are approximately 8 kb and 3 kb upstream of *IL-28B* gene, respectively. Because these SNPs, which showed the greatest statistical significance in the previous study, are located far from the *IL-28B* gene, another approach was required to determine the effect of the SNPs. In this study, broad (TA)_n_ variations were observed in rs8099917 heterozygotes among CHC patients. Interestingly, a combination of TG and 11/12 genotype was strongly associated with NVR, whereas patients harboring the 12/13 genotype showed a virological response, regardless of the TG genotype (rs8099917). In clinical practice, genetic diagnosis using TA variation, following the primary classification of rs8099917 genotype, could improve the prediction of treatment response for CHC patients with the rs8099917 TG genotype. It is not clear whether the variation originates from genetic or epigenetic mechanisms. In addition, as the frequency of TA variation might be dependent on the particular population, further study will be needed to compare the frequency in several populations. A long TA repeat, over (TA)_13_, was observed in healthy volunteers and showed potential for higher gene expression compared with under (TA)_13_ constructs *in vitro*. It may be possible that spontaneous clearance of HCV infection and CHC patients are affected by this region because this also is dependent on *IL-28B* genotype [15], [19]. In our speculation, the combination of both TA variation and the landmark SNPs, rs8099917 and rs12979860, might improve the prediction value. In addition, convenient diagnosis method to detect the TA variation like SNPs typing is needed since the present capillary techniques are relative complexity compared with SNPs typing.

In the international database, some SNPs ID are registered in the TA repeat region, located in the regulatory regions of the *IL-28A* and *IL-28B* gene, rs72284792 and rs7225881, respectively, whereas in our analysis separating *IL-28A* from *IL-28*, TA variation was detected only in the *IL-28B* region. SNP data often have been collected using next generation sequencing and based on short sequence reads. Unfortunately, the sequence similarity between *IL-28A* and *IL-28B* is over 90% from the CpG island to the region downstream of 3′-UTR. Alignment failure would occur for a high percentage of sequences when analyzed with software using general algorithms.

Effects of insertion/deletion (indel) polymorphism are known in the field of pharmacogenetic research. A polymorphism in the promoter of the uridine diphosphoglucuronosyl transferase 1A1 (*UGT 1A1*) gene has been shown to cause Crigler-Najjar syndrome types I and II and Gilbert syndrome, a benign form of unconjugated hyperbilirubinemia, and the occurrence of severe toxic event**s** in irinotecan (known as CPT-11) administration [24], [25], [26]. The polymorphism consists of a (TA)_n_ repeat in the 5′-promoter region [24], [26], [27], similar to that in this study. The range of repeat numbers is from (TA)_5_ to (TA)_8_ in the *UGT 1A1* gene [28]. The genetic disorder of the TA repeat length affects enzyme activity. The hepatic bilirubin *UGT 1A1* activity of individuals with Gilbert's syndrome is <30% of normal [29]. Irinotecan is used or under evaluation for a broad spectrum of solid tumors. Irinotecan pharmacokinetic parameters display a wide inter-patient variability and are involved in the genesis of toxic side effects [30], [31], [32], [33]. Based on the polymorphism of the TA repeat, previous papers reported the association of irinotecan-induced severe toxicity with Gilbert's syndrome [34], [35], [36]. The value of genetic diagnosis of the *UGT1A1* polymorphisms prior to irinotecan chemotherapy has been corroborated in a previous study [37]. As similar characteristics were observed in the upstream region of *IL-28B*, the (TA)_n_ repeat might be associated with disease progression as well as response to anti-HCV treatment.

In terms of epigenetic aspects, the TA variation of *IL-28B* was also suspected to be related to microsatellite instability, because a gap between the significant SNPs and TA variation was observed in this study. DNA mismatch repair (MMR) deficiency causes a high frequency of microsatellite instability (MSI-H), which is characterized by length alterations within simple repeated sequences, microsatellites. Lynch syndrome is primarily due to germline mutations in one of the DNA MMR genes, hMLH1 or hMSH2 [38]. MSI-H is also observed in <15% of colorectal, gastric and endometrial cancers, where it is associated with the hypermethylation of the promoter region of hMLH1 [39], [40]. The diagnosis of MSI-H in cancers is therefore useful for identifying patients with Lynch syndrome and the efficacy of chemotherapy [41], [42], [43], [44], [45], [46].

In conclusion, a (TA) dinucleotide repeat, rs72258881, located in the promoter region, was discovered by our functional studies of the proximal SNPs around *IL-28B*; the transcriptional activity of the promoter increased gradually in a (TA)_n_ length-dependent manner. Combination diagnosis based on rs8099917 and rs72258881 might provide improved prediction because the (TA)_n_ variation of *IL-28B* was observed but not that of *IL-28A*. The further study is needed to reveal the association with treatment response using clinical specimens of CHC. These findings suggest that the dinucleotide repeat could be associated with the transcriptional activity of *IL-28B* as well as constituting a predictor to improve prediction of the response to interferon-based HCV treatment.

## Supporting Information

Figure S1 **Sequence alignment of** ***IL-28A/B*** **cDNA retrieved from the database.** The cDNA sequences of *IL-28A/B* were retrieved from the international database using accession number. The cDNA data reported by Sheppard et al. are AY129148 (*IL-28A*) and AY129149 (*IL-28B*) indicated with ‘_S’ in the figure, and that of Kotenko et al. are AY184373 (*IL-28A*) and AY184374 (*IL-28B*) indicated with ‘_K’. Dashed boxes show the start codon predicted by computational analysis of the human genome reported by Sheppard et al. and Kotenko et al. The sequence alignment was calculated with Lasergene software (DNASTAR, Madison, WI).
(PDF)

Figure S2 **Structural similarity between** ***IL-28A*** **and** ***IL-28B*** **.** (A) Schematic of *IL-28A/B* gene location (UCSC genome browser). Boxes show the region representing high levels of structural similarity around *IL-28A/B*. (B) Modified schematic of structural similarity with a percentage. (C) Alignment between IL-28A and IL-28B from the CpG island to the region downstream of 3′-UTR. Homologous regions are shown by red characters. High levels of structural similarity were observed in CpG island, regulatory and gene region bypassing the in/del site.
(PDF)

Figure S3 **Innate immune receptor expression related to IL-28B regulation.** The relevant receptors for this study were confirmed by PCR using specific primers. (A) The mRNA expression of TLR4 was detected in cell lines, HeLa, Jurkat, MT-2, Raji, and PBMC. (B) For the study of cytokine-receptor association, the expression of IL-28RA and IL-10RB second receptor were examined using cDNA obtained from HuH7, HepG2, and HuSE2 cells. Samples without reverse transcriptase were prepared as a negative control in addition to the checking of genome contamination.
(PDF)

Figure S4 **Direct sequencing analysis of TA repeat.** In the first step to determine (TA)_n_ genotypes, direct sequencing was applied to amplicons of *IL-28A* or *28B* separated by gel electrophoresis. Homozygotes of TA repeat showed clear patterns and a high quality value in the bar above, whereas the patterns of heterozygotes were mixed because the length differed between alleles. The mixed patterns are shown in dashed boxes. These mixed products were cloned into the pGEM-Teasy vector to isolate and count the (TA)_n_ number by sequencing of both alleles.
(PDF)

Table S1 (DOC)

Table S2 (DOC)

Table S3 (DOC)

Table S4 (DOC)
