# Supplementary material for: Genetic Variation of the IL-28B Promoter Affecting Gene Expression
Source: PLoS One. 2011 Oct 25;6(10):e26620. doi: 10.1371/journal.pone.0026620 (PMC3201970; doi:10.1371/journal.pone.0026620)
Supplement: Figure S1 — Sequence alignment of IL-28A/B cDNA retrieved from the database. The cDNA sequences of IL-28A/B were retrieved from the international database using accession number. The cDNA data reported by Sheppard et al. are AY129148 (IL-28A) and AY129149 (IL-28B) indicated with ‘_S’ in the figure, and that of Kotenko et al. are AY184373 (IL-28A) and AY184374 (IL-28B) indicated with ‘_K’. Dashed boxes show the start codon predicted by computational analysis of the human genome reported by Sheppard et al. and Kotenko et al. The sequence alignment was calculated with Lasergene software (DNASTAR, Madison, WI). (PDF) [file pone.0026620.s001.pdf]

```
IL28A_S.seq ATGAAACTAGACATGACTGGGGACTGCACGCCAGTGCTGGTGCTGATGGCCGCAGTGCTG    60
IL28A_K.seq ------------ATGACTGGGGACTGCACGCCAGTGCTGGTGCTGATGGCCGCAGTGCTG    48
IL28B_S.seq ATGAAACTAGACATGACCGGGGACTGCATGCCAGTGCTGGTGCTGATGGCCGCAGTGCTG    60
IL28B_K.seq ------------ATGACCGGGGACTGCATGCCAGTGCTGGTGCTGATGGCCGCAGTGCTG    48

IL28A_S.seq ACCGTGACTGGAGCAGTTCCTGTCGCCAGGCTCCACGGGGCTCTCCCGGATGCAAGGGGC    120
IL28A_K.seq ACCGTGACTGGAGCAGTTCCTGTCGCCAGGCTCCACGGGGCTCTCCCGGATGCAAGGGGC    108
IL28B_S.seq ACCGTGACTGGAGCAGTTCCTGTCGCCAGGCTCCGCGGGGCTCTCCCGGATGCAAGGGGC    120
IL28B_K.seq ACCGTGACTGGAGCAGTTCCTGTCGCCAGGCTCCGCGGGGCTCTCCCGGATGCAAGGGGC    108

IL28A_S.seq TGCCACATAGCCCAGTTCAAGTCCCTGTCTCCACAGGAGCTGCAGGCCTTTAAGAGGGCC    180
IL28A_K.seq TGCCACATAGCCCAGTTCAAGTCCCTGTCTCCACAGGAGCTGCAGGCCTTTAAGAGGGCC    168
IL28B_S.seq TGCCACATAGCCCAGTTCAAGTCCCTGTCTCCACAGGAGCTGCAGGCCTTTAAGAGGGCC    180
IL28B_K.seq TGCCACATAGCCCAGTTCAAGTCCCTGTCTCCACAGGAGCTGCAGGCCTTTAAGAGGGCC    168

IL28A_S.seq AAAGATGCCTTAGAAGAGTCGCTTCTGCTGAAGGACTGCAGGTGCCACTCCCGCCTCTTC    240
IL28A_K.seq AAAGATGCCTTAGAAGAGTCGCTTCTGCTGAAGGACTGCAGGTGCCACTCCCGCCTCTTC    228
IL28B_S.seq AAAGATGCCTTAGAAGAGTCGCTTCTGCTGAAGGACTGCAAGTGCCGCTCCCGCCTCTTC    240
IL28B_K.seq AAAGATGCCTTAGAAGAGTCGCTTCTGCTGAAGGACTGCAAGTGCCGCTCCCGCCTCTTC    228

IL28A_S.seq CCCAGGACCTGGGACCTGAGGCAGCTGCAGGTGAGGGAGCGCCCCATGGCTTTGGAGGCT    300
IL28A_K.seq CCCAGGACCTGGGACCTGAGGCAGCTGCAGGTGAGGGAGCGCCCCATGGCTTTGGAGGCT    288
IL28B_S.seq CCCAGGACCTGGGACCTGAGGCAGCTGCAGGTGAGGGAGCGCCCCGTGGCTTTGGAGGCT    300
IL28B_K.seq CCCAGGACCTGGGACCTGAGGCAGCTGCAGGTGAGGGAGCGCCCCGTGGCTTTGGAGGCT    288

IL28A_S.seq GAGCTGGCCCTGACGCTGAAGGTTCTGGAGGCCACCGCTGACACTGACCCAGCCCTGGTG    360
IL28A_K.seq GAGCTGGCCCTGACGCTGAAGGTTCTGGAGGCCACCGCTGACACTGACCCAGCCCTGGTG    348
IL28B_S.seq GAGCTGGCCCTGACGCTGAAGGTTCTGGAGGCCACCGCTGACACTGACCCAGCCCTGGGG    360
IL28B_K.seq GAGCTGGCCCTGACGCTGAAGGTTCTGGAGGCCTCCGCTGACACTGACCCAGCCCTGGGG    348

IL28A_S.seq GACGTCTTGGACCAGCCCCTTCACACCCTGCACCATATCCTCTCCCAGTTCCGGGCCTGT    420
IL28A_K.seq GACGTCTTGGACCAGCCCCTTCACACCCTGCACCATATCCTCTCCCAGTTCCGGGCCTGT    408
IL28B_S.seq GATGTCTTGGACCAGCCCCTTCACACCCTGCACCATATCCTCTCCCAGCTCCGGGCCTGT    420
IL28B_K.seq GATGTCTTGGACCAGCCCCTTCACACCCTGCACCATATCCTCTCCCAGCTCCGGGCCTGT    408

IL28A_S.seq ATCCAGCCTCAGCCCACGGCAGGGCCCAGGACCCGGGGCCGCCTCCACCATTGGCTGTAC    480
IL28A_K.seq ATCCAGCCTCAGCCCACGGCTGGGCCCAGGGCCCGGGGCCGCCTCCACCATTGGCTGTAC    468
IL28B_S.seq ATCCAGCCTCAGCCCACGGCAGGGCCCAGGACCCGGGGCCGCCTCCACCATTGGCTGCAC    480
IL28B_K.seq ATCCAGCCTCAGCCCACGGCAGGGCCCAGGACCCGGGGCCGCCTCCACCATTGGCTGTAC    468

IL28A_S.seq CGGCTCCAGGAGGCCCCAAAAAAGGAGTCCCCTGGCTGCCTCGAGGCCTCTGTCACCTTC    540
IL28A_K.seq CGGCTCCAGGAGGCCCCAAAAAAGGAGTCCCCTGGCTGCCTCGAGGCCTCTGTCACCTTC    528
IL28B_S.seq CGGCTCCAGGAGGCCCCAAAAAAGGAGTCCCCTGGCTGCCTCGAGGCCTCTGTCACCTTC    540
IL28B_K.seq CGGCTCCAGGAGGCCCCAAAAAAGGAGTCCCCTGGCTGCCTCGAGGCCTCTGTCACCTTC    528

IL28A_S.seq AACCTCTTCCGCCTCCTCACGCGAGACCTGAATTGTGTTGCCAGTGGGGACCTGTGTGTC    600
IL28A_K.seq AACCTCTTCCGCCTCCTCACGCGAGACCTGAATTGTGTTGCCAGTGGGGACCTGTGTGTC    588
IL28B_S.seq AACCTCTTCCGCCTCCTCACGCGAGACCTGAATTGTGTTGCCAGCGGGGACCTGTGTGTC    600
IL28B_K.seq AACCTCTTCCGCCTCCTCACGCGAGACCTGAATTGTGTTGCCAGCGGGGACCTGTGTGTC    588

IL28A_S.seq TGA                                                             603
IL28A_K.seq TGA                                                             591
IL28B_S.seq TGA                                                             603
IL28B_K.seq TGA                                                             591
```

Figure S1
