# Supplementary figures and images for: Genetic Variation of the IL-28B Promoter Affecting Gene Expression
Source: PLoS One. 2011 Oct 25;6(10):e26620. doi: 10.1371/journal.pone.0026620 (PMC3201970; doi:10.1371/journal.pone.0026620)

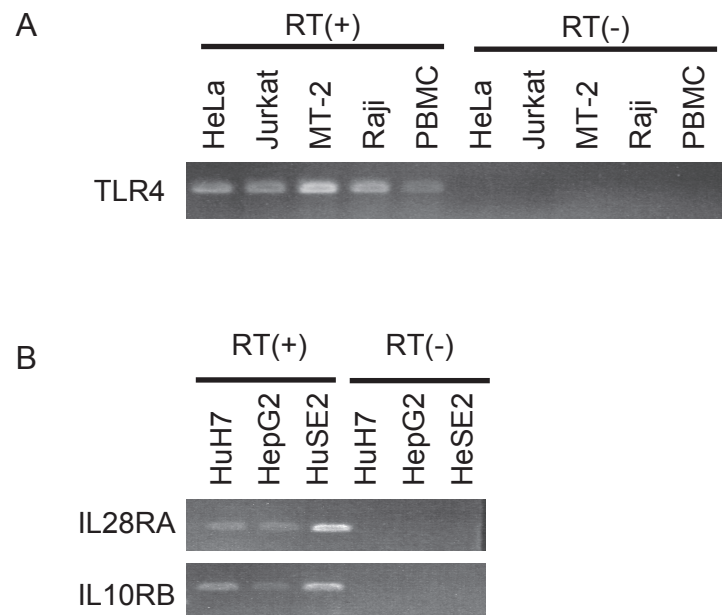

Figure S3

Supplement: Figure S3 — Innate immune receptor expression related to IL-28B regulation. The relevant receptors for this study were confirmed by PCR using specific primers. (A) The mRNA expression of TLR4 was detected in cell lines, HeLa, Jurkat, MT-2, Raji, and PBMC. (B) For the study of cytokine-receptor association, the expression of IL-28RA and IL-10RB second receptor were examined using cDNA obtained from HuH7, HepG2, and HuSE2 cells. Samples without reverse transcriptase were prepared as a negative control in addition to the checking of genome contamination. (PDF) [file pone.0026620.s003.pdf]

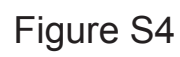

Figure S4

Supplement: Figure S4 — Direct sequencing analysis of TA repeat. In the first step to determine (TA)n genotypes, direct sequencing was applied to amplicons of IL-28A or 28B separated by gel electrophoresis. Homozygotes of TA repeat showed clear patterns and a high quality value in the bar above, whereas the patterns of heterozygotes were mixed because the length differed between alleles. The mixed patterns are shown in dashed boxes. These mixed products were cloned into the pGEM-Teasy vector to isolate and count the (TA)n number by sequencing of both alleles. (PDF) [file pone.0026620.s004.pdf]
