# Supplementary material for: Genetic Variation of the IL-28B Promoter Affecting Gene Expression
Source: PLoS One. 2011 Oct 25;6(10):e26620. doi: 10.1371/journal.pone.0026620 (PMC3201970; doi:10.1371/journal.pone.0026620)
Supplement: Table S1 — (DOC) [file pone.0026620.s005.doc]

| Primer |  | Sequence |
| --- | --- | --- |
| Expression | F1 | CACCATGAAACTAGGTGAGTCCCAC |
|  | R2 | TCCGACACACAGGTCCCCGCTGGC |
| RACE | P1 | CACTTGCAGTCCTTCAGCAG |
|  | P2 | TCTTTGGCCCTCTTAAAGGC |
|  | P3 | CTGCACCGGCTCCAGGAGGCCCCAAAAAAG |
| Genotyping | G1 | CTTCCTTGCCTGGGCAATTAAGAAATATTG |
|  | G2 | AAGCTTCGACAGAGATGTGGGACTCACCTAGTTTC |
|  | Seq1 | CCCAAGAGGATTCCACCTGCTCTGG |
|  | Seq2 | GGGGCAGCTTTTATCCCTGACAGAA |
| qPCR | FLAG/F | GCTTATCGATACCGTCGACCT |
|  | FLAG/R | GGGCGAATTGGGTACACTTA |
|  | GAPDH/F | CACCAGGGCTGCTTTTAACTC |
|  | GAPDH/R | AGATGGTGATGGGATTTCCA |
| PCR | TLR4/F | GAAGGGGTGCCTCCATTTCA |
|  | TLR4/R | ACCAGCGGCTCTGGATGAAG |
|  | IL28RA/F | ACCTATTTTGTGGCCTATCAGAGCT |
|  | IL28RA/R | CGGCTCCACTTCAAAAAGGTAAT |
|  | IL10RB/F | AGGGCTGAATTTGCAGATGA |
|  | IL10RB/R | CCGTTTTTCCAGTATTGCAC |

Table S1. Primer sequences

The underlined nucleotide was changed to avoid a stop codon for the expression of the tagged protein.
