# Supplementary material for: Genetic Variation of the IL-28B Promoter Affecting Gene Expression
Source: PLoS One. 2011 Oct 25;6(10):e26620. doi: 10.1371/journal.pone.0026620 (PMC3201970; doi:10.1371/journal.pone.0026620)
Supplement: Table S2 — (DOC) [file pone.0026620.s006.doc]

Table S2. Statistical analysis of Fig. 3B

|  | WW | MW | WM | MM | CTRL |
| --- | --- | --- | --- | --- | --- |
| WW | - | < 0.05 | 0.162 | < 0.05 | < 0.05 |
| MW |  | - | < 0.05 | < 0.05 | < 0.05 |
| WM |  |  | - | < 0.05 | < 0.05 |
| MM |  |  |  | - | < 0.05 |
| CTRL |  |  |  |  | - |
