# Supplementary material for: Genetic Variation of the IL-28B Promoter Affecting Gene Expression
Source: PLoS One. 2011 Oct 25;6(10):e26620. doi: 10.1371/journal.pone.0026620 (PMC3201970; doi:10.1371/journal.pone.0026620)
Supplement: Table S3 — (DOC) [file pone.0026620.s007.doc]

Table S3. Statistical analysis of Fig. 3D

|  | WW | WM | MW | MM |
| --- | --- | --- | --- | --- |
| WW | - | < 0.05 | 0.415 | < 0.05 |
| WM |  | - | < 0.05 | 0.484 |
| MW |  |  | - | < 0.05 |
| MM |  |  |  | - |
