# Supplementary material for: Genetic Variation of the IL-28B Promoter Affecting Gene Expression
Source: PLoS One. 2011 Oct 25;6(10):e26620. doi: 10.1371/journal.pone.0026620 (PMC3201970; doi:10.1371/journal.pone.0026620)
Supplement: Table S4 — (DOC) [file pone.0026620.s008.doc]

Table S4. Statistical analysis of Fig. 6

|  | 10 | 11 | 12 | 13 | 14 | 15 | 16 | 17 | 18 |
| --- | --- | --- | --- | --- | --- | --- | --- | --- | --- |
| 10 | - | < 0.05 | < 0.05 | < 0.05 | < 0.05 | < 0.05 | < 0.05 | < 0.05 | < 0.05 |
| 11 |  | - | < 0.05 | < 0.05 | < 0.05 | < 0.05 | < 0.05 | < 0.05 | < 0.05 |
| 12 |  |  | - | < 0.05 | < 0.05 | < 0.05 | < 0.05 | < 0.05 | < 0.05 |
| 13 |  |  |  | - | 0.120 | 0.132 | 0.016 | < 0.05 | < 0.05 |
| 14 |  |  |  |  | - | 0.631 | 0.083 | < 0.05 | < 0.05 |
| 15 |  |  |  |  |  | - | 0.241 | < 0.05 | < 0.05 |
| 16 |  |  |  |  |  |  | - | < 0.05 | < 0.05 |
| 17 |  |  |  |  |  |  |  | - | 0.219 |
| 18 |  |  |  |  |  |  |  |  | - |
