# Supplementary material for: Genetic Variation of the IL-28B Promoter Affecting Gene Expression
Source: PLoS One. 2011 Oct 25;6(10):e26620. doi: 10.1371/journal.pone.0026620 (PMC3201970; doi:10.1371/journal.pone.0026620)
Supplement: Figure S2 — Structural similarity between IL-28A and IL-28B . (A) Schematic of IL-28A/B gene location (UCSC genome browser). Boxes show the region representing high levels of structural similarity around IL-28A/B. (B) Modified schematic of structural similarity with a percentage. (C) Alignment between IL-28A and IL-28B from the CpG island to the region downstream of 3′-UTR. Homologous regions are shown by red characters. High levels of structural similarity were observed in CpG island, regulatory and gene region bypassing the in/del site. (PDF) [file pone.0026620.s002.pdf]

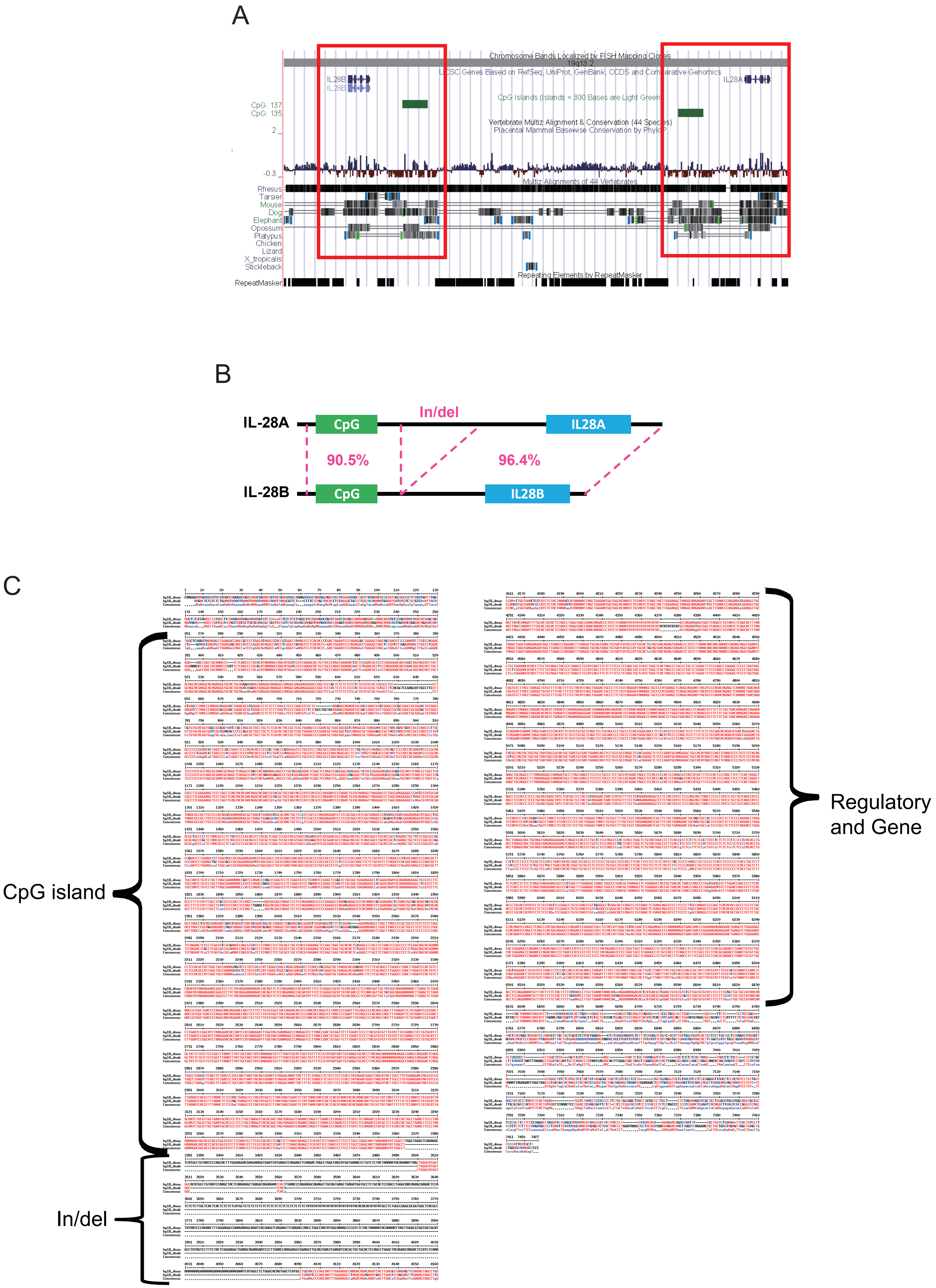

A
Chromosome Bands Localized by FISH Mapping Clones
19q13.2
UCSC Genes Based on RefSeq, UniProt, GenBank, CCDS and Comparative Genomics
IL28B
IL28A
CpG Islands (Islands < 300 Bases are Light Green)
CpG: 137
CpG: 135
Vertebrate Multiz Alignment & Conservation (44 Species)
Placental Mammal Basewise Conservation by PhyloP
Multiz Alignments of 44 Vertebrates
Rhesus
Tarsier
Mouse
Dog
Elephant
Opossum
Platypus
Chicken
Lizard
X_tropicalis
Stickleback
Repeating Elements by RepeatMasker
RepeatMasker
B
IL-28A
IL-28B
CpG
In/del
IL28A
IL28B
90.5%
96.4%
C
CpG island
In/del
Regulatory and Gene

Figure S2
